# Global perspectives on *Klebsiella* epidemiology and biology: conference report on the KLEBS 2024 symposium

**DOI:** 10.1038/s44259-025-00175-3

**Published:** 2026-01-27

**Authors:** Chiara Crestani, Kelly L. Wyres, Jabir Abdulahi, Archana Angrup, William Boateng, Chanté Brand, Arsène G. Djoko Nono, Teca C. Galvao, Devarshi Gajjar, Francisco Gonzalez-Espinosa, Mateusz Hasso-Agopsowicz, Yogesh Hooda, Sanika M. Kulkarni, Rudzani Mashau, Richael O. Mills, Geetha Nagaraj, Issa Ndiaye, Courtney P. Olwagen, Lala Rafetrarivony, Andriniaina Rakotondrasoa, Denasha L. Reddy, Varun Shamanna, Liliwe Shuping, Talyta Soares do Nascimento, Blessing K. A. Tabi, Lara Van der Merwe, Kathryn E. Holt, Sylvain Brisse

**Affiliations:** 1 Institut Pasteur, Université Paris Cité, Biodiversity and Epidemiology of Bacterial Pathogens Paris, Paris, France; 2 Monash University, Melbourne, VIC Australia; 3 London School of Hygiene & Tropical Medicine, London, UK; 4 Postgraduate Institute of Medical Education and Research, Chandigarh, India; 5 Noguchi Memorial Institute for Medical Research, Accra, Ghana; 6 Stellenbosch University, Cape Town, South Africa; 7 Centre Pasteur du Cameroun, Yaoundé, Cameroon; 8 Fundação Oswaldo Cruz, Rio de Janeiro, Brazil; 9 The Maharaja Sayajirao University of Baroda, Vadodara, India; 10 University of Buenos Aires, Consejo Nacional de Investigaciones Científicas y Técnicas (CONICET), Buenos Aires, Argentina; 11 World Health Organization (WHO), Geneva, Switzerland; 12 Child Health Research Foundation, Dhaka, Bangladesh; 13 Christian Medical College, Vellore, India; 14 Wits Mycology Division, School of Pathology, Faculty of Health Sciences, University of the Witwatersrand, Johannesburg, South Africa; 15 University of Cape Coast, Cape Coast, Ghana; 16 Central Research Laboratory-KIMS, Bengaluru, India; 17 Institut Pasteur Dakar, Dakar, Senegal; 18 Institut Pasteur de Madagascar, Antananarivo, Madagascar; 19 Institut Pasteur de Bangui, Bangui, Central African Republic; 20 National Institute for Communicable Diseases, a Division of the National Health Laboratory Service, Johannesburg, South Africa

**Keywords:** Diseases, Health care, Medical research, Microbiology

## Abstract

The inaugural *Klebsiella* Epidemiology and Biology Symposium (KLEBS) took place in November 20-22, 2024, at the Institut Pasteur in Paris. It covered a broad multidisciplinary range of topics from fundamental biology to public health aspects, including epidemiology and public health burden, One Health and clinical aspects, genomics, host-pathogen interactions, vaccines, and therapeutics. This report describes research presented during keynote presentations, plenary sessions and a panel discussion. A recording of the conference is available at: https://www.klebs-2024.conferences-pasteur.org/replay.

## Introduction

**Fig. 1:**
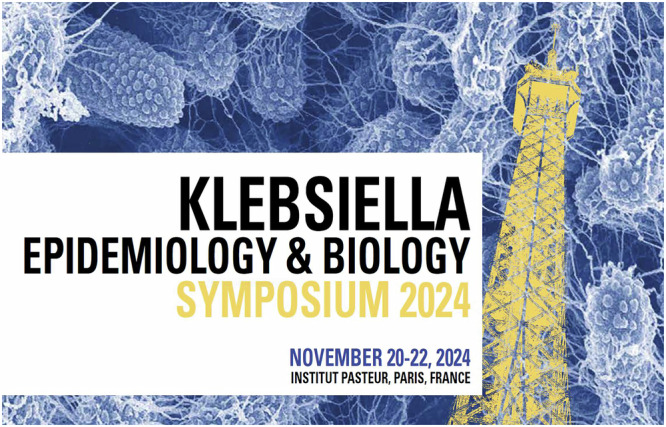
The *Klebsiella* Epidemiology and Biology Symposium (KLEBS) 2024 conference banner.

*Klebsiella*, and particularly *Klebsiella pneumoniae* (Kpn), has emerged as one of the leading causes of nosocomial infections globally, and it is notorious for its multidrug resistance. Given the serious public health threat it represents, due to the rising resistance rates to multiple antibiotic classes, the risk of convergence of genetic determinants of antimicrobial resistance and hypervirulence traits, and the current lack of a vaccine, Kpn was recently placed at the top position of priority pathogens by the World Health Organization (WHO). A scientific event dedicated to *Klebsiella* was long overdue. The first Klebsiella Epidemiology and Biology Symposium (KLEBS) took place in Paris in 2024 (Fig. 1). The hybrid format of the event aimed to promote inclusion and knowledge sharing, attracting 320 on-site participants and a cumulative virtual audience of 650 over the three days, from 71 countries. To facilitate global participation, 40 travel grants were awarded to selected presenters from low- and middle-income countries (LMICs). The program included 42 oral communications and 216 posters, some of which were selected for 1-min pitch presentations. Additionally, a pre-conference workshop on genomic surveillance analytical tools was co-organized by the KlebNET consortium for 50 participants. Recordings of most presentations are available at: https://www.klebs-2024.conferences-pasteur.org/replay.

## Opening session

Following a welcome address by Sylvain Brisse (Institut Pasteur, Paris, France), Kathryn Holt (London School of Hygiene & Tropical Medicine, London, United Kingdom) presented a comprehensive review of 15 years of Kpn research in her keynote address. She traced the journey from sequencing the first Kpn genome in 2009^1^ to the nearly 75,000 genomes publicly available today, underscoring the pivotal role of genomics in population biology and understanding the impact of gene transfer, local to global transmission, outbreak investigation and epidemiological surveillance^2^. Holt emphasized the increasing recognition of Kpn’s clinical significance, primarily due to its alarming accumulation of antimicrobial resistance (AMR) genes and the emergence of hypervirulent strains (hvKpn) carrying plasmids and mobile elements with siderophores and adhesins^3^. The dissemination of high-risk clones, such as carbapenem-resistant sequence type (ST) 258, is a major contributor to AMR-associated deaths globally. In addition, convergence of virulence and resistance traits within the same strains is particularly concerning. Genomic studies have also revealed Kpn’s considerable genetic diversity, population structure, evolutionary dynamics, pathogenicity, and ecology. Key genomic tools that have significantly contributed to these findings include Kleborate^4^ developed by her research group, and Kaptive^5,6^ developed in collaboration with Kelly Wyres’s group (Monash University, Melbourne, Australia). Lastly, she discussed the challenges associated with developing polyvalent vaccines to address Kpn’s surface antigen diversity, in particular to inform vaccines preventing Kpn neonatal sepsis in LMICs, and called for global collaboration through initiatives like KlebNET (https://klebnet.org) to combat this growing threat using shared genomic data and innovative interventions.

The second invited speaker in this session was Shabir Madhi (University of the Witwatersrand, Johannesburg, South Africa), who shared his insights on the clinical and molecular epidemiology of invasive Kpn using data from Africa and South Asia. He highlighted diagnostic limitations in accurately capturing Kpn’s actual contribution to neonatal mortality, as demonstrated in meta-analyses and post-mortem findings^7,8^. While neonates experience a higher incidence of infection, the absolute disease burden is three to four times greater in adults, particularly among those with comorbidities such as human immunodeficiency virus (HIV) and diabetes, emphasizing the urgent need for vaccines^9,10^, he also cautioned against the growing threat of AMR.

Next, Christian Giske (Karolinska Institute, Stockholm, Sweden) addressed the burden and management of Kpn in Europe and the Americas. He reaffirmed Kpn’s disproportionate impact on patients with comorbidities, significant burden in neonatal and hospital-acquired infections, and rising resistance rates, with a 57.8% increase in carbapenem resistance between 2019 and 2023 in European countries^11^. He introduced the main genetic drivers of AMR in Kpn, including metallo-β-lactamase- and carbapenemase-encoding genes such as *bla*_KPC_, *bla*_NDM_, and *bla*_OXA-48_, as well as emerging mutations in penicillin-binding proteins (PBPs). He reminded the audience of concerns regarding hvKpn strains that have acquired multi-drug resistance, such as ST23^12^, highlighting the limited treatment options due to rising resistance rates to antibiotics like colistin and new cephalosporin/beta-lactamase inhibitor combination agents. He concluded by discussing promising alternatives, including bacteriophage therapy (see Section “Vaccines and novel therapeutic approaches”), while emphasizing the need for more research in these areas.

Collectively, these presentations underscore the critical necessity for global efforts to combat Kpn through advanced genomic tools, innovative treatments, and vaccine development.

## Epidemiology in humans

This session focused on the clinical and molecular epidemiology of *Klebsiella* infections across diverse geographic regions, including Asia, South America, Europe, and Africa, offering a comprehensive view of AMR and hvKpn.

With regards to Asia, Quynh Nguyen (Oxford University Clinical Research Unit, Ho Chi Minh, Vietnam) presented results from a study on hvKpn in Vietnam^13^, finding a prevalence of 14.3% among bloodstream isolates and 3.7% among rectal swabs from healthy adults. hvKpn isolates from both sources belonged to the same phylogenetic clusters, suggesting that human intestinal carriage may play a role in hvKpn transmission. The isolates exhibited significant genetic diversity, encompassing 17 STs (the most common being ST23, ST86, and ST65). Furthermore, these isolates demonstrated the ability to easily acquire virulence markers such as *iuc1* and *iuc3* through plasmid conjugation, emphasizing their adaptability.

Fernando Gonzalez-Candelas (University of Valencia, Valencia, Spain) presented a genomic study of over 1700 clinical isolates from Spain, identifying distinct transmission patterns driving the spread of third-generation cephalosporin- (3GC) and carbapenem-resistant Kpn. Resistance to 3GC involved a complex interplay between intra-hospital and inter-hospital transmission, primarily linked with lineage ST307 and the *bla*_CTX-M-15_ gene, which disseminated across hospitals through intermediate community steps. In contrast, resistance to carbapenems emerged within diverse genetic backgrounds and genes in each hospital, primarily spreading through intra-hospital dissemination^14^.

In Bangladesh, a longitudinal study presented by Yogesh Hooda (Child Health Research Foundation, Bangladesh) detected a concerning rise in carbapenem-resistant isolates among 1700 Kpn collected over an 18-year period and primarily driven by dissemination of *bla*_NDM-1_. There was a simultaneous increase in mortality (from 30% before 2015 to 52% in 2021), but this was not driven directly by the increasing carbapenem resistance rates. Whole genome sequencing (WGS) for ~520 isolates revealed high genetic diversity and showed shifts in dominant lineages throughout the study period.

Caroline Tigoi, who presented on behalf of Wilson Gumbi (KEMRI-Wellcome Trust Research Programme, Kilifi, Kenya), shared findings from the NeoBAC study in Kenya. The study, conducted in three hospitals, included 403 pediatric isolates and 27 environmental samples. Retrospective genomic analyses revealed multiple outbreaks of the ST14 clone that were previously undetected during routine care, highlighting the potential of genomic surveillance in recognizing nosocomial outbreaks. While known for being resistant to carbapenems, ST14 strains in this study exhibited resistance to cephalosporins (*bla*_OXA-1_ and *bla*_CTX-M-15_) but not to carbapenems. A different ST (ST39) was predominant among environmental samples, indicating that the environment may not significantly contribute to invasive infections in these hospitals.

In India, Geetha Nagaraj (Kempegowda Institute of Medical Sciences, Bangalore, India) reported that carbapenem-resistant Kpn isolates exhibited changing patterns in resistance genes over six years (2014–2022), with *bla*_NDM_ prevalence increasing from 7.8% to 59.3% and *bla*_OXA-48_ prevalence decreasing from 91.3% to 73%^15^. These changes, along with the co-occurrence of resistance genes, illustrate the complex and evolving AMR landscape in the region.

Similar trends were observed in Chile, where the Chilean Public Health Institute reported a marked increase in Kpn isolates resistant to carbapenems between 2014 and 2019^16^. Among various carbapenem-resistant clones, an ST23 isolate co-producing *bla*_KPC-2_ and *bla*_VIM-1_ on a highly transmissible conjugative plasmid was identified by Andreas Marcoleta (Universidad de Chile, Santiago, Chile) and colleagues^17^. This plasmid likely evolved locally from a *bla*_KPC-2_ plasmid widely disseminated in South America and linked to a carbapenem-resistant multispecies outbreak in Chile^18^, after acquiring an integron carrying the *bla*_VIM-1_ gene. The study also observed a high prevalence of clonal group (CG) 10224 (ST25, K2), an originally carbapenem-susceptible clone, but now resistant^16^. These results illustrate the usefulness of genomic analysis in AMR surveillance both locally and globally.

### *Klebsiella pneumoniae* in LMICs: the path toward a maternal vaccine (reports from the Gates Foundation Partners)

This session was organized by the Gates Foundation, the main sponsor of the KLEBS 2024 conference, and was moderated by Nicole Benson and Usman Nakakana. It highlighted the burden of neonatal sepsis caused by Kpn in LMICs and explored progress in maternal vaccine development as a potential solution. Neonatal sepsis is a significant cause of mortality, particularly in regions such as Africa and Southeast Asia, where hospital-acquired infections and AMR are prevalent.

Ziyaad Dangor (University of the Witwatersrand, Johannesburg, South Africa) presented data from the Child Health and Mortality Prevention and Surveillance study (CHAMPS)^8^, showing that Kpn contributed to 18% of neonatal deaths and 24% of deaths among children under five years old, with preterm infants and malnourished children being particularly vulnerable. These findings underscore the urgent need for preventive strategies, such as multivalent vaccines targeting the capsule (K antigen) or the LPS (lipopolysaccharide, O antigen), a challenge given the high diversity of surface antigens in *K. pneumoniae*. Prof Dangoor highlighted that preclinical studies on glycoconjugate and outer membrane vesicle-based vaccine platforms demonstrated promising immune responses against both K- and O-antigens, although challenges remain in achieving broad cross-reactivity across diverse serotypes.

Francesca Micoli (GSK Vaccines Institutes for Global Health, Siena, Italy) detailed progress in developing vaccine platforms targeting Kpn antigens such as K-antigen, O-antigen, and MrkA (type 3 fimbriae). Glycoconjugate vaccines exhibited high consistency through CDAP (1-cyano-4-dimethylaminopyridine tetrafluoroborate) chemistry, ensuring reliable conjugation of polysaccharide antigens to carrier proteins. Additional platforms included a multiple antigen presentation system (MAPS) technology for conjugating biotinylated polysaccharides, outer membrane vesicles for presenting antigens on strains undergoing blebbing, and nanoparticles for showcasing K- and O-antigens alongside MrkA. Preclinical studies revealed that both anti-KAg and anti-OAg antibodies elicited strong binding and killing with some level of cross-reactive responses. However, there was strain-to-strain variability. In contrast, anti-MrkA antibodies demonstrated lower binding and immune activity. Challenges persist in optimizing cross-reactivity and ensuring robust immune responses across diverse Kpn serotypes.

Heather Fox (Inventprise Inc., Redmond, United States) presented the development of glycoconjugate vaccines specifically targeting Kpn serotypes identified in CHAMPS data. A 5-valent formulation included K2, K25, K102, O1, and O5 antigens, while a 10-valent formulation added K15, K62, O2afg, O3b, and O4 for expanded coverage. Both formulations used rCRM197 (derived from diphtheria toxin) as a carrier protein and alum as an adjuvant to enhance immunogenicity. Efficacy studies using a rabbit vaccination model demonstrated strong serotype-specific opsonophagocytic activity for four tested antigens, with further evaluation ongoing for the fifth antigen. Fox highlighted the potential to produce high-quality, low-cost vaccines to address the pressing need for effective and scalable solutions in LMICs.

Collectively, the session underscored the promises of vaccine approaches in preventing Kpn-associated neonatal sepsis and early childhood deaths. The presented approaches demonstrated innovative vaccine platforms and promising preclinical results, laying the groundwork for future clinical trials.

## *Klebsiella* ecology and one health

Like other ESKAPE pathogens which are ecological generalists, Kpn has a broad distribution beyond its human association, which has motivated studies into the relationships and transmission of Kpn populations with a One Health perspective. This session explored the genomic diversity, AMR profiles, and transmission dynamics across human, animal, and environmental reservoirs.

Marit Hetland (Stavanger University Hospital, Stavanger, Norway) presented data from a study analyzing over 3000 Kpn isolates collected over 20 years from three ecological niches: humans, animals, and the environment^19^. The Kpn Species Complex (KpSC) populations across these niches were found to be distinct yet not entirely separate, with limited but observable zoonotic spillover events. Approximately 5% of human infection isolates were closely related to those from animal or marine sources, despite time and location separation. Regarding AMR, most resistance genes were identified in human isolates, with carbapenemases and extended-spectrum beta-lactamases (ESBLs) predominantly detected in clinical settings. The study further highlighted the presence of multidrug-resistant clones (such as ST15 and ST307) and virulence factors like aerobactin in animals, alongside the spillover of relevant virulence plasmids between pigs and humans^19^. Genome-wide association analyses identified over 40 niche-enriched traits and significant genetic diversity across the niches, with specific AMR genes particularly enriched in human isolates.

In the following talk, Lala Rafetrarivony (Pasteur Institute of Madagascar, Antananarivo, Madagascar) presented results from the SARA project (Surveillance of Antimicrobial Resistance in Africa), a multi-country collaborative initiative aimed at enhancing AMR genomic surveillance and research in Africa^20^. Third generation cephalosporin resistant isolates were collected from three niches following the WHO Tricycle protocol^21^: humans (pregnant women, *n* = 289), animals (chickens, *n* = 730), and the environment (surface water, *n* = 96). The study revealed a high prevalence of ESBL-producing KpSC in pregnant women (20%), associated with multidrug resistance reaching around 45% in humans and exceeding 60% in non-human isolates, confirming Kpn’s prominent role as an indicator organism for monitoring AMR across sectors.

Marisa Haenni (French Agency for Food, Environmental and Occupational Health & Safety, ANSES, Lyon, France) presented data on the emergence of carbapenem-resistant Kpn isolates in companion animals in France. Utilizing a national veterinary AMR surveillance network spanning over 100 laboratories (Résapath, https://resapath.anses.fr/) and having operated across a ten-year period (2014–2023), the study identified 87 carbapenem-resistant isolates belonging to various STs (most common were ST11, ST15, ST307). All isolates harbored the *bla*_OXA-48_ gene, which is also the main driver of resistance to carbapenems in the French human population, on highly transmissible IncL plasmids^22,23^. Most of these isolates were multidrug-resistant and detected AMR genes were generally linked to resistance to first-line antibiotics used for therapy in companion animals (e.g., amoxicillin/penicillin, aminoglycosides, sulphonamides, tetracyclines). She also showed how some strains were suspected to be responsible for outbreaks in certain veterinary clinics, with less than 10 single-nucleotide polymorphisms between them^24^.

Lesley Hoyles (Nottingham Trent University, Nottingham, United Kingdom) presented research on *Klebsiella* species previously classified under the *Raoultella* genus (*Klebsiella planticola* complex, KplC, and *Klebsiella terrigena* complex, KtC). The study analyzed more than 1,000 genomes, revealing that KplC and KtC share significant phylogenetic and functional similarities with Kpn and unveiled novel beta-lactamase variants (*bla*_PLA_, *bla*_ORN_, *bla*_TER_). The presented data supported the reclassification of KplC and KtC within the *Klebsiella* genus and underscored the potential of synthetic biology in elucidating the functional diversity of chromosomal beta-lactamases in this group.

Finally, José Delgado-Blas presented an extensive analysis of the genome dynamics of KpSC across four ecological niches: wastewater (*n* = 31), human clinical samples (*n* = 47), cattle farms (*n* = 304), and garden market farms (*n* = 329) sampled in a single region of France over one year. Among the 336 KpSC isolates, Kpn was predominant and demonstrated high genetic diversity and niche adaptation, particularly in clinical settings, with 19% classified as high-risk sublineages, and extensive AMR genes content. *Klebsiella variicola* subsp. *variicola* exhibited environmental specialization and a larger core genome. AMR genes and plasmids were most prevalent in clinical and wastewater isolates, emphasizing ecological compartmentalization. Long-term evolutionary divergence may have shaped Kpn for human environments, while *K. variicola* subsp. *variicola* adapted to environmental niches.

Together, the findings reveal a complex and interlinked *Klebsiella* ecology where distinct yet intersecting human, animal, and environmental populations show the exchange of resistance and virulence determinants, underscoring the importance of One Health-based genomic surveillance to capture these connections and inform AMR control efforts.

## *Klebsiella* molecular biology

The emergence of hvKpn strains, especially those exhibiting multidrug resistance, has raised significant global health concerns. This session explored the molecular mechanisms underlying Kpn’s virulence and adaptability, focusing on how environmental factors, genetic backgrounds, and specific molecular interactions contribute to its pathogenicity.

The session began with Alix Lee (Queen’s University Belfast, Belfast, United Kingdom), who presented findings on Type VI Secretion Systems (T6SS). The study revealed that Kpn strains exhibit T6SS behaviors that are either offensive (targeting competitors) or defensive (activated in response to aggressive competitors). The regulation of T6SS behavior is influenced by a single amino acid polymorphism in the *Klebsiella* T6SS Regulator A protein sequence, which governs the transcriptional expression of the T6SS. Furthermore, a significant correlation was identified between defensive T6SS behavior and AMR profiles, with defensive T6SS possibly promoting the spread of AMR and virulence factors.

Next, Yanjie Chao (Shanghai Institute of Immunity and Infection, Shanghai, China) presented a study using a novel intracellular RNA interactome profiling method (iRIL-seq) to identify over 30 small RNAs (sRNAs) that interact with capsule biosynthesis genes^25^. In a mouse model with nasal infection and subsequent dissemination of Kpn to the lungs, spleen and liver, ArcZ emerged as a crucial regulator of virulence by repressing capsule-related genes (*mlaA* and *fbp*) through direct base-pairing interactions. ArcZ is transcriptionally activated by the catabolite regulator CRP and is part of a novel regulatory circuit controlling hypermucoviscosity and virulence (CRP-ArcZ-MlaA). Overexpression of ArcZ reduced capsule production, bacterial burden, and hypermucoviscosity in multiple carbapenem-resistant and hvKpn clinical isolates, highlighting its potential therapeutic application as an RNA inhibitor in bacterial pneumonia.

Michael Bachman (Michigan University, Ann Arbor, United States) focused on Kpn bacteremia, revealing novel insights into bacterial dissemination within hosts. Using a barcoding approach (STAMPR, Sequence-Tagged Analysis of Microbial Populations in R)^26^, Bachman and his team tracked the movement of Kpn from the lungs to secondary organs during pneumonia. Their findings identified two distinct patterns of dissemination: ‘metastatic’, characterized by clonal expansion in the lungs followed by spread to secondary sites (spleen, liver, blood) with high similarity, and ‘direct’, characterized by immediate exit from the lungs without clonal expansion, resulting in lower systemic burdens and greater dissimilarity from lung isolates^26^. These patterns were influenced by bacterial fitness and host immune responses, presenting a new paradigm for understanding within-host bacterial spread and emphasizing the heterogeneity of dissemination dynamics.

Julie Le Bris (Institut Pasteur, Paris, France) discussed findings demonstrating how capsule type K64 significantly influences mucoviscosity and metabolic traits, leading to notable effects on cell physiology and fitness trade-offs, such as reduced resistance to bile salts. By introducing K64 into diverse genetic backgrounds, including hypervirulent and commensal strains, the research uncovered negative epistasis and host genome-dependent effects on capsule production and maintenance. K64 strains exhibited increased plasmid acquisition in hypervirulent backgrounds, facilitating the convergence of resistance and hypervirulence^27^. This research highlights the genetic interplay between serotype-specific factors and the bacterial genome, emphasizing the need to target such mechanisms to control the spread of carbapenem-resistant hvKpn strains.

Finally, Laura Mike (University of Pittsburgh, Pittsburgh, United States) presented research on how discrete environmental cues regulate capsule production and mucoidy in Kpn strains. The study revealed that sugar import reduces mucoidy by decreasing *rmpD* transcription, while specific amino acids like arginine increase mucoidy by upregulating *rmpD*^28^. These regulatory alterations affect capsular polysaccharide chain length and uniformity through RmpD’s interaction with Wzc, a regulator of capsular gene length. Using RNA-seq and transposon mutagenesis, Mike’s team identified regulatory pathways mediating these effects. They also demonstrated how these cues influence Kpn’s interactions with host cells. The findings shed light on Kpn’s ability to integrate environmental signals to optimize niche-specific fitness, providing new insights into the mechanisms underlying hypermucoviscosity and virulence.

Altogether, the session highlighted how Kpn integrates genetic, regulatory, and environmental signals to fine‑tune its virulence, antibiotic resistance, and dissemination, underscoring the complex interplay between molecular mechanisms and ecological context in the emergence of hvKpn.

### Host–pathogen interactions

This session showcased cutting-edge research on Kpn virulence mechanisms, emphasizing its adaptability and interactions with host cells. Presentations covered diverse topics, including the role of T6SS effectors, immune evasion factors, mucosal pathogenesis, capsule heterogeneity, and the genetic transition from classical Kpn (cKpn) to hvKpn.

Jose Bengoechea (Queen’s University Belfast, Belfast, United Kingdom) presented work from his group on the development of an infection model using rat arteries to investigate the impact of Kpn on vasodilation. This work explored whether Kpn exploits the trans-kingdom effector VgrG4, found in ~11% Kpn, to manipulate host cells^29^. The group discovered that VgrG4 induces calcium transfer from the endoplasmic reticulum to mitochondria, activating the GTPase dynamin-related protein 1 (Drp1) for mitochondrial fragmentation and NLRX1 (a mitochondrial nucleotide-binding oligomerization domain-like, or NOD-like, receptor) activation^30^. Additionally, their work revealed a new anti-host role of T6SS, showing how Kp manipulates vascular biology by targeting post-translational modifications of eNOS (endothelial nitric oxide synthase)^31^.

KivA (*Klebsiella* immune evasin A) is a virulence factor contributing to immune evasion in Kpn. Joana Sá-Pessoa (Queen’s University Belfast, Belfast, United Kingdom) and her colleagues demonstrated that Kpn delivers KivA into mammalian cells through T6SS. KivA manipulates host responses and blocks toll-like receptor (TLR) signaling induced by various agonists. A *kivA* mutant induced more inflammation in vitro and in vivo and exhibited attenuated virulence in *Galleria mellonella* and mouse pneumonia models. Notably, KivA was found to be both necessary and sufficient to increase the virulence of a Kpn strain lacking the KivA protein, which was associated with a reduced inflammatory response.

Teck Hui Teo (A*STAR ID Labs, Singapore, Singapore) presented findings on the differential mucosal pathogenesis of cKpn ST258 versus hvKpn ST23 in respiratory infections^32^. The authors suggest that variability in disease outcomes could be due to differential interaction between myeloid cells and cKpn or hvKpn. While neutrophils were found to be protective in infections caused by cKpn, they exacerbated inflammation in hvKpn infections. The authors proposed that targeting neutrophil functions, such as NETosis, adhesion, and protease activity, might represent a novel host-directed therapy against hvKpn.

Joseph Wanford (King’s College London, London, United Kingdom) investigated hvKpn’s capacity for phase variation, a trait that enables pathogens to adapt to fluctuating environments. Alongside his colleagues, he demonstrated high frequency, reversible mutations at simple sequence repeats in the *rmp* locus mediate combinatorial ON/OFF variation of capsule expression and mucoidy in K1 and K2 strains. Notably, OFF variants were associated with lower serum survival rates but greater epithelium invasion. The development of an in vitro assay to measure population-wide mutations in *rmp* simple sequence repeats, showed that repeat tract expansion was a key driver of capsule heterogeneity during non-selective culture.

The final talk in this session was presented by Stephen Salisbury (University of North Carolina, Chapel Hill, United States), whose research focused on mechanisms driving the transition from cKpn to hvKpn. He introduced the *rmpADC* operon with its native promoter into the chromosome of cKpn. This *rmpADC* introduction conferred a hypermucoviscous phenotype^33^, increased capsule production, and enhanced virulence-associated traits in cKpn strains. Hypermucoviscous-positive classical strains with *rmpADC* genes were less adherent to epithelial and immune cells and demonstrated greater resistance to phagocytosis by J774 macrophage cells. However, not all hypermucoviscous-positive strains exhibited increased virulence in mouse pneumonia models, and further analysis is warranted to identify the genetic requirements for the acquisition of hypermucoviscosity and hypervirulence.

Overall, the session highlighted the multifaceted nature of Kpn virulence, revealing how diverse molecular strategies, from immune evasion and vascular manipulation to phase variation and genetic recombination, collectively underpin its adaptability, pathogenic potential, and transition between classical and hypervirulent forms.

## Infection models

This session was dedicated to infection models, which are essential tools for studying pathogens, including Kpn, and which enable researchers to investigate virulence mechanisms, host–pathogen interactions and the clinical impacts of antimicrobial resistance.

The session began with two invited speakers. First, Ammar Zafar (Wake Forest University School of Medicine, Winston-Salem, United States) presented his research on hvKpn, highlighting aerobactin’s key role in Kpn translocation from the gastrointestinal tract to sterile sites. Utilizing both murine and in vitro models, including a model of colonisation and inter-host transmission, Zafar and his team demonstrated that the absence of aerobactin impairs iron homeostasis, leading to decreased adhesion, invasion, and translocation, ultimately resulting in reduced virulence^34^. These findings reinforce the view that aerobactin is a central factor in hvKpn’s pathogenicity and suggest it could serve as a promising target for therapeutic approaches.

David Rosen (Washington University, St. Louis, United States) followed with a presentation focusing on the application of murine models to explore the pathogenesis, immunity, and therapeutics of Kpn. He emphasized the relevance of these models due to their anatomical and immunological similarities to humans, as well as their genetic malleability and cost-effectiveness. Rosen reviewed various murine models, including those designed for urinary tract infections^35^, pneumonia^36^, and bacteremia (e.g., mostly via intraperitoneal injection or tail vein injection). He demonstrated how initial studies on bioconjugate capsule vaccines targeting K1 and K2 elicited robust immune responses and conferred protection in murine models^37^. He also showed data suggesting that capsule-based vaccines may be superior to O-antigen vaccines for targeting hvKp and some cKp strains, due to the capsule blocking antibody access to the O-antigen^38^. Rosen highlighted how murine models, despite inherent limitations, remain essential for advancing therapies and vaccines against Kpn.

Sarah Rowe-Conlon (University of North Carolina at Chapel Hill, Chapel Hill, United States) addressed antibiotic treatment failure in liver abscesses caused by hvKpn, highlighting the role of antibiotic-tolerant persister cells in poor treatment outcomes. These cells survive antibiotics due to low adenosine triphosphate (ATP) levels, particularly in stationary-phase bacteria. To investigate whether antibiotic tolerance can contribute to treatment failure in Kpn infections, Rowe-Conlon and her team used a novel mouse hvKp wound infection model^39^. They found that antibiotics were effective only in the early stages of infection, with persister cells contributing to treatment failure. Importantly, treatment failure against liver abscesses was not associated with the development of antibiotic resistance, suggesting a role for antibiotic tolerant cells.

Ricardo Calderón González (Queen’s University Belfast, Belfast, United Kingdom) presented a novel in vivo approach utilizing Bac-CyTOF mass cytometry to track immune interactions during Kpn pneumonia^40^. Over a 72-h infection period, the study observed progressive neutrophilia, increased inflammatory monocytes and interstitial macrophages, and a reduction in alveolar macrophages, which are typically effective against infections. The T6SS shifted bacterial interactions from alveolar to interstitial macrophages, suppressing efficient immune responses and promoting bacterial survival. These findings highlight Kpn’s capacity to manipulate the immune environment to favor less effective cell populations, limiting host defenses.

The session underscored how diverse in vivo and in vitro infection models can provide critical insights into Kpn pathogenesis, host interactions, and treatment responses, thereby driving the development of more effective therapeutics and preventive strategies.

## Genomics and evolution

This session showcased advancements in the genomic surveillance and evolution of Kpn, focusing on capsule swapping, metabolic modeling, and plasmid-driven resistance and virulence. It highlighted global monitoring efforts, outbreak dynamics, and the evolution of resistance genes that shape Kpn’s public health impact.

The session featured two invited speakers, beginning with David Aanensen (Oxford University, Oxford, United Kingdom). He outlined the approaches used in genomic surveillance projects, including EuSCAPE (European Survey of Carbapenemase-Producing Enterobacteriaceae) in European countries^41^ and GHRU (Global Health Research Unit) in LMICs^42^, which aim to enhance local and international data collection to identify, monitor, and combat the spread of resistant bacteria threatening public health. The EuSCAPE survey has led to an additional follow-up survey by the European Centre for Disease Prevention and Control (ECDC) to track ST39, noting an increased proportion of carbapenem resistance detected during the study period^43^. He emphasized the importance of sentinel sites for strain collection and the necessity of strengthening the capabilities of LMICs in genomic surveillance through collaborative efforts. Aanensen also introduced public platforms developed by the Centre for Genomic Pathogen Surveillance (CGPS, https://www.pathogensurveillance.net/), such as Pathogenwatch (https://pathogen.watch/)^44^, Microreact (https://microreact.org/)^45^, and AMRwatch (https://amr.watch/), describing how these can enhance genomic surveillance globally.

Olaya Rendueles (Centre National de la Recherche Scientifique, CNRS, Toulouse, France), the second invited speaker, discussed how Kpn evolution may be driven by the swapping of capsular polysaccharides among different lineages. These polysaccharides are critical structural components of the capsule, influencing its physical and chemical properties and thereby determining how the bacterium interacts with its environment, host immune defenses, and phages. Rendueles and her colleagues discovered that the sugar molecules in capsules undergoing swapping are more genetically similar to each other than those in capsules that do not swap^27^. Additionally, she highlighted the role of extracellular capsules, which are key determinants of phage infection, in Kpn evolution and their repercussions. The capsular serotype change in Kpn significantly impacts gene flow via plasmids, as well as phage-induced gene flow, with capsule evolution shaping the Kpn species and affecting traits like hypermucoviscosity and biofilm formation^46^.

Ben Vezina (Monash University, Melbourne, Australia) and colleagues studied metabolic diversity within the KpSC using comparative genomics and genome-scale metabolic modeling to explain lineage diversity. They hypothesized that metabolic differentiation drives lineage-specific behaviors, enabling adaptation to diverse environments and hosts. Analyzing over 7800 genomes, they leveraged the curated a KpSC pan metabolic model and employed Bactabolize to predict metabolic networks and growth phenotypes for individual isolates^47^. Their analysis revealed that sublineages possess distinct metabolic profiles, with no pair sharing the same set of core metabolic traits. These metabolic differences facilitate commensal interactions, reduce competition, and support the coexistence of diverse sublineages^48^.

Melissa Martin (Walter Reed Army Institute of Research, Silver Spring, United States) presented findings from a decade-long study on Kpn and the plasmidome. A retrospective analysis of genomes from 1472 multidrug-resistant Kpn isolates from the Multidrug-Resistant Organism Repository and Surveillance (MRSN, https://wrair.health.mil/Biomedical-Research/Center-for-Infectious-Disease-Research/Bacterial-Diseases/Multidrug-Resistant-Organism-Repository-and-Surveillance-Network/) revealed a convergence of resistance and virulence driven by the acquisition of separate AMR and virulence plasmids, alongside hybrid plasmids carrying both gene types. Notably, three independent sublineages of ST395 emerged simultaneously in Georgia, each acquiring distinct AMR and virulence plasmids rather than inheriting them from a common ancestor. Comparative plasmid and phylogenetic analyses showed that these events involved separate plasmid incompatibility groups, confirming multiple, independent convergence events. This illustrates the value of integrated genomic and plasmidomic surveillance approaches for distinguishing local plasmid transmission from parallel evolution across lineages.

Ana Weber (Charité – Universitätsmedizin Berlin, Germany) and colleagues reported a rise in *bla*_NDM-1_-producing *Klebsiella pneumoniae* (Kpn) in Germany after 2022, possibly linked to population movement during the conflict in Ukraine^49^. Genomic analysis of 71 NDM-producing Kpn isolates from four Berlin hospitals revealed three outbreak clusters (O1–O3) involving ST147 and ST395. Using a combination of short- and long-read sequencing with hybrid assembly and plasmid clustering (MOB-suite; https://github.com/oschwengers/tadrep), the team reconstructed *bla*_NDM_ plasmids and identified two distinct plasmid clusters. Smaller, non-mobilizable IncFIB plasmids (52–54 kb) were found in ST147 from hospitals A and B (O1), which larger conjugative IncFIB plasmids (351–355 kb) were associated with ST147 and ST395 in other sites (O2–O3). Genetic differences between these plasmids ruled out a single multi-site plasmid outbreak, highlighting once again the utility of plasmidomics for resolving complex transmission scenarios.

Lastly, Evan Snitkin (University of Michigan, Ann Arbor, United States) and his team investigated genetic and clinical factors influencing resistance in Kpn ST258 across a network of 11 long-term acute care hospitals in Los Angeles. They identified three distinct clades, with the emergence and spread of resistance varying between them. Colistin resistance emerged independently 40 times but was predominantly retained in clade IIB due to its association with respiratory infections and increased colistin exposure. In contrast, imipenem-relebactam resistance was more prevalent in clade I, facilitated by a lineage-specific *ompK36* mutation interacting with a *bla*_KPC_-carrying plasmid. These findings underscore the role of genetic background in the evolution of resistance.

The session underscored the power of integrated genomic, metabolic, and plasmidomic approaches to better understand the evolutionary forces shaping Kpn diversity, demonstrating how global surveillance can help tracking capsule dynamics, plasmid exchange, and the dissemination of resistant and hypervirulent clones.

## Vaccines and novel therapeutic approaches

This session focused on innovative strategies to combat Kpn infections through vaccine development, monoclonal antibodies, and bacteriophage therapies.

The invited speaker for this session, Sara Federici (Weizmann Institute of Science, Rehovot, Israel), presented research on phage therapy targeting Kpn strains associated with inflammatory bowel disease (IBD). A cohort of over 500 patients across four countries underwent metagenomic sequencing, revealing a strong association between Kpn (specifically Kp2-clade ST323 strains) and severe IBD symptoms^50^. In germ-free mouse models, Kp2-clade strains induced inflammation by lowering IL-10 (interleukin-10) levels and increasing interferon-gamma production. To counteract antibiotic resistance, the team developed a phage cocktail specifically targeting Kp2-clade strains. In vitro studies demonstrated effective suppression of Kpn growth, while in vivo tests showed reduced inflammation and improved survival. A phase 1 trial involving 18 healthy individuals confirmed the persistence of the phages in the gut for six days post-administration without altering microbiome composition, underscoring phage therapy as a precise and safe alternative to antibiotics in managing IBD^50^.

Paeton Wantuch (Washington University, Saint Louis, United States) reported on a broad-spectrum capsule bioconjugate vaccine (Omniose, Saint Louis, Mo, United States), targeting serotypes K1, K2, K102, and K107, designed to protect against both hvKpn and cKpn. A prime-boost-boost immunization regimen in CD1 mice using a 1 µg polysaccharide dose elicited robust IgG responses across all four capsular types. SBA (Serum Bactericidal Activity) and OPKA (Opsonophagocytic Killing Assay) assays confirmed potent bactericidal activity against multiple Kpn strains. In vivo, the vaccine significantly improved survival in a murine bacteremia model, especially against hvKpn. To enhance the protection effect against classical isolates, a neutropenic mouse model using cyclophosphamide was developed, resulting in a two-log reduction of the infectious dose and near-complete protection. Functional antibody responses were maintained for at least six months post-immunization.

Juan Jose Infante (VAXDYN, Dos Hermanas, Spain) introduced K-Vax, a whole-cell vaccine utilizing *Acinetobacter baumannii* as a carrier for conserved outer membrane proteins (OMP) OmpA and OmpK36. The vaccine conferred protection in murine models of sepsis and pneumonia against both hvKpn and cKpn strains. Functional antibodies generated by the vaccine opsonized bacterial cells, facilitating internalization and destruction by human phagocytes, regardless of capsule presence or OMP variability. In silico analysis using the CARB-X database suggested broad strain coverage. These findings highlight the potential of this candidate vaccine for further clinical development, including trials for at-risk adults and maternal immunization to prevent neonatal sepsis.

Anna Kabanova (Fondazione Toscana Life Sciences, Siena, Italy) presented the development of potent anti-capsular human monoclonal antibodies (mAbs) aimed at combating hypervirulent, pandrug-resistant Kpn ST147 (Roscioli et al.^51^). Through antigen-agnostic screening of memory B cells from convalescent donors, the study identified 20 bactericidal mAbs targeting three pathogenic serotypes. Two distinct clusters of mAbs emerged—one targeting the K64 capsule and the other targeting the O2a antigen^51^. In vitro assays demonstrated picomolar-range bactericidal activity, though in vivo assessments revealed that only KL64 mAbs offered protection against severe ST147 bloodstream infections. This protection was linked to enhanced macrophage uptake and bacterial chain formation, inhibiting proliferation^51^. The research underscores an innovative approach to developing therapeutic mAbs for emerging multidrug-resistant Kpn strains.

Daniela Rothschild Rodriguez (University of Southampton, Southampton, United Kingdom) introduced KlebPhaCol (https://www.klebphacol.org/), an open-source community-driven platform that provides access to fully characterized bacteriophages targeting *Klebsiella* spp.^52^. The platform currently hosts 53 phages and 74 clinical strains of *Klebsiella* spp., encompassing 40 STs, 32 capsular types, and 10 O-antigens, with an emphasis on Kpn. The phages encompass five different phage families, one of which is a novel phage family proposed as *Felixviridae*, six known genera, the three main morphotypes and are all but two, virulent phages. Novel phages, Roth C and Roth D, are included in the collection. KlebPhaCol facilitates studies on phage-host interactions, development of phage therapies, and efficacy assessments against antibiotic-resistant *Klebsiella* strains. Researchers can contribute new isolates, analyze genomic data, and compare phage susceptibility across strains, fostering collaboration and accelerating the discovery and application of bacteriophage therapies for *Klebsiella* infections.

Overall, this session offered comprehensive insights into cutting-edge therapeutic approaches targeting Kpn, emphasizing the importance of an integrated strategy combining vaccine development, monoclonal antibody therapies, and phage innovations to address the challenges posed by AMR infections.

## Panel discussion: how do you solve a problem like Klebsiella? Focus on surveillance and infection control

The panel discussion brought together leading experts to address the pressing challenges posed by Kpn infections. Moderated by Padmini Srikantiah (Gates Foundation, Seattle, United States), the discussion centered on how current research can enhance surveillance systems and infection control measures for Kpn.

Kathryn Holt (London School of Hygiene & Tropical Medicine, London, United Kingdom) emphasized the transformative role of WGS in tracking outbreaks and understanding the evolving genetic diversity of Kpn. She called for the integration of genomic surveillance into routine public health practices rather than restricting it to research settings, highlighting the critical importance of metadata. The panel agreed that integrating clinical and genomic data is essential for designing and implementing control strategies.

Michael Bachman (University of Michigan, Ann Arbor, United States) discussed the challenges associated with diagnosing infections and advocated for the development of advanced diagnostics to improve treatment timelines. He stressed the importance of expanding surveillance to include not only KpSC but also other *Enterobacteriaceae*, which involves robust culture data and the identification of risk factors for infection.

Amy Mathers (University of Virginia School of Medicine, Charlottesville, United States) focused on the rise of carbapenem-resistant Kpn, arguing that antimicrobial stewardship and reliable diagnostics are key priorities to curb transmission, with hospital environmental screening playing a role in surveillance.

Shabir Madhi (University of the Witwatersrand, Johannesburg, South Africa) provided a perspective from LMICs, emphasizing the burden of neonatal *Klebsiella* infections and the limited access to new antibiotics. He underscored the significance of understanding and preventing invasive diseases, noting that one-third of infections are polymicrobial and that the contributions of individual species or synergistic interactions among them remain underexplored. Both Madhi and other panelists advocated for careful evaluation of microbiome-targeted interventions.

On the topic of vaccine development, Keith Klugman (Gates Foundation, Seattle, United States) acknowledged the scientific and financial obstacles presented by *Klebsiella*’s continuously evolving strain diversity. He proposed maternal immunization as a potential solution, but both he and Shabir Madhi highlighted its limitations, particularly regarding polymicrobial infections. The panel emphasized the need for flexible, regionally tailored vaccine platforms and proof-of-concept studies to evaluate novel approaches, such as K-antigen-based vaccines.

The panel also discussed the importance of basic infection prevention measures, including hygiene practices, handwashing, and environmental cleaning, which remain critical across all settings. Expanding access to diagnostics, implementing antimicrobial stewardship programs, and enhancing environmental surveillance and colonization screening were highlighted as foundational components in the fight against resistance.

In conclusion, the panel emphasized the importance of integrating genomic surveillance, enhanced diagnostics, infection control measures, and vaccine development into a multifaceted approach to tackle Kpn. Collaborative global efforts and investments were deemed crucial for overcoming the challenges posed by antimicrobial resistance and improving public health outcomes.

## Conclusions

The 1st *Klebsiella* Epidemiology and Biology Symposium featured a broad overview of cutting-edge research on the microbiology of *Klebsiella* and its interactions with the host, and featured significant insights into the current challenges and advancements in tackling Kpn infections on a global scale. The presentations and discussions underscored the urgent need for collaborative initiatives across various sectors, including research, healthcare, and public health.

An emphasis was placed on innovative approaches combining genomic surveillance, vaccine development, and novel therapeutic strategies, such as phage therapy and monoclonal antibodies. These solutions aim to address the increasing threat posed by multidrug-resistant Kpn, especially in vulnerable populations across LMICs.

The symposium also highlighted the critical need for ongoing monitoring of Kpn lineages evolution and resistance patterns, as well as the importance of global collaboration in sharing data and resources to effectively manage this public health challenge. Continued investments into research, development, and implementation of prevention strategies will be essential to diminish the clinical burden of Kpn and safeguard public health in communities worldwide. As we move forward, fostering partnerships and encouraging interdisciplinary dialogue will be vital in mobilizing the resources and knowledge necessary to confront the challenges posed by Kpn infections. The KLEBS 2024 conference was a first step in gathering a multidisciplinary research community around this prominent pathogen, creating multiple novel professional interactions that will promote the acceleration of research and the development of novel control measures against *Klebsiella*. There was a unanimous feeling among the participants that the conference was a remarkable scientific and social success that will hopefully be reproduced within the next few years.

## Supplementary information

Supplementary Information

## Data Availability

No datasets were generated or analyzed during the current study.
