# Supplementary material for: Global perspectives on Klebsiella epidemiology and biology: conference report on the KLEBS 2024 symposium
Source: NPJ Antimicrob Resist. 2026 Jan 27;4:4. doi: 10.1038/s44259-025-00175-3 (PMC12847830; doi:10.1038/s44259-025-00175-3)
Supplement: Supplementary file 1 — Supplementary Information [file 44259_2025_175_MOESM1_ESM.pdf]

Supplementary material

Global perspectives on *Klebsiella* Epidemiology and Biology: conference report on the KLEBS 2024 symposium

Crestani Chiara[1], Wyres Kelly L[2,3], Abdulahi Jabir[3], Angrup Archana[4], Boateng William[5], Brand Chanté[6], Djoko Nono Arsène G[7], Galvao Teca C[8], Gajjar Devarshi[9], Gonzalez-Espinosa Francisco[10], Hasso-Agopsowicz Mateusz[11], Hooda Yogesh[12], Kulkarni Sanika M[13], Mashau Rudzani[14], Mills Richael O[15], Nagaraj Geetha[16], Ndiaye Issa[17], Olwagen Courtney P[14], Rafetrarivony Lala[18], Rakotondrasoa Andriniaina[19], Reddy Denasha L[14], Shamanna Varun[16], Shuping Liliwe[20], Soares do Nascimento Talyta[8], Tabi Blessing K A[5], Van der Merwe Lara[14], Holt Kathryn E[2,3], and Brisse Sylvain[1*].

Affiliations:

1. Institut Pasteur, Université Paris Cité, Biodiversity and Epidemiology of Bacterial Pathogens Paris, France
2. Monash University, Melbourne, Australia
3. London School of Hygiene & Tropical Medicine, London, UK
4. Postgraduate Institute of Medical Education and Research, Chandigarh, India
5. Noguchi Memorial Institute for Medical Research, Accra, Ghana
6. Stellenbosch University, Cape Town, South Africa
7. Centre Pasteur du Cameroun, Yaoundé, Cameroon
8. Fundação Oswaldo Cruz, Instituto Oswaldo cruz, Rio de Janeiro, Brazil
9. The Maharaja Sayajirao University of Baroda, Vadodara, India
10. University of Buenos Aires, Consejo Nacional de Investigaciones Científicas y Técnicas (CONICET), Buenos Aires, Argentina
11. World Health Organization (WHO), Geneva, Switzerland
12. Child Health Research Foundation, Dhaka, Bangladesh
13. Christian Medical College, Vellore, India
14. Wits Mycology Division, School of Pathology, Faculty of Health Sciences, University of Witwatersrand, Johannesburg, South Africa
15. University of Cape Coast, Cape Coast, Ghana
16. Central Research Laboratory-KIMS, Bengaluru, India
17. Institut Pasteur Dakar, Dakar, Senegal
18. Institut Pasteur de Madagascar, Antananarivo, Madagascar
19. Institut Pasteur de Bangui, Bangui, Central African Republic
20. National Institute for Communicable Diseases, a Division of the National Health Laboratory Service, Johannesburg, South Africa

***Correspondence:** Prof Sylvain Brisse, Institut Pasteur, Biodiversity and Epidemiology of Bacterial Pathogens, 25-28 rue du Docteur Roux, F-75724, Paris, France; Phone: +33 1 45 68 83 34; E-mail: sylvain.brisse@pasteur.fr. ORCID: 0000-0002-2516-2108.

Conference programme

Klebsiella Epidemiology and Biology Symposium 2024

November 20th-22nd, 2024
Institut Pasteur, Paris, France

Wednesday, November 20th, 2024

PRE-SYMPOSIUM WORKSHOP

A pre-symposium workshop on KlebNET tools for *Klebsiella* strain characterization and genomes bioinformatics analytics took place from 9:00 to 12:00 on Nov. 20th in the Auditorium.

1:00-1:15 pm - Welcome address

Sylvain Brisse, Conference Chair

1:15-3:00pm - Opening session

Chairs: Sylvain Brisse, Institut Pasteur, France - Chiara Crestani, Institut Pasteur, France

- Genomic insights into Kpn biology and epidemiology

  Invited speaker: **Kat Holt** (London School of Hygiene & Tropical Medicine, London, United Kingdom)

- Clinical and molecular epidemiology of invasive *Klebsiella pneumoniae*: An African experience

  Invited speaker: **Shabir Madhi** (Vaccines and Infectious Diseases Analytics Research Unit, University of The Witwatersrand, Johannesburg, South Africa)

- Burden and management of Kpn infection: an American/European perspective

  Invited speaker: **Christian Giske** (Karolinska Institute, Stockholm, Sweden)

3:30-5:30pm - Session 1: Epidemiology in humans

Chairs: Kat Holt, London School of Hygiene & Tropical Medicine, United Kingdom - Arnfinn Sundsfjord, The Arctic University of Norway, Norway

- Genomic insights unveil the plasmid transfer mechanism and epidemiology of hypervirulent *Klebsiella pneumoniae* in Vietnam

  Selected abstract: **Quynh Nguyen** (Oxford University Clinical Research Unit, HCMC, Vietnam, Ho Chi Minh, Vietnam)

- Different transmission patterns between 3GC- and carbapenem-resistant *Klebsiella pneumoniae*

  Selected abstract: **Fernando Gonzalez-Candelas** (Joint Research Unit Infection and Public Health, University of Valencia - FISABIO, Valencia, Spain)

- Escalating burden and mortality of *Klebsiella* infections in young infants in Bangladesh: a prospective genomic epidemiology study over 18 years

  Selected abstract: **Yogesh Hooda** (Child Health Research Foundation, Dhaka, Bangladesh)

- Whole-Genome Sequencing reveals potential *Klebsiella pneumoniae* ST14 outbreaks in Kiambu County and Mbagathi Subcounty Hospitals of Kenya in 2021 and 2022 respectively

Selected abstract: **Caroline Tigoi** (presenting on behalf of Wilson Gumbi, Bioscience Department, KEMRI-Wellcome Trust Research Programme, Kilifi, Kenya)

- Trends of Clonal Lineage Evolution and Emergence Among Carbapenem-Resistant *K. pneumoniae* in Indian Hospital Environments

  Selected abstract: **Geetha Nagaraj** (CRL, KIMS, Bangalore, India)

- High-risk *K. pneumoniae* clones and mobile elements in Chile and South America: empowering the National AMR Surveillance with genomics

  Selected abstract: **Andres Marcoleta** (Grupo de Microbiologia Integrativa, Facultad de Ciencias, Universidad de Chile, Santiago, Chile)

5:30-5:50 pm - Poster pitches

Chair: Chiara Crestani, Institut Pasteur, France

Giglione B., Genetic Diversity of *Klebsiella* spp. from Wastewater in Argentina: Towards the Standardization of AMR Monitoring for Wastewater-Based Surveillance in Hospital Settings

Paranagama K., Metabolic diversity of *Klebsiella pneumoniae* and association with ecological niche

Wall K., Herbicide-induced effects on the transcriptome, metabolome and proteome of *Klebsiella* species

Fernandes L., Genomic characterization of carbapenemase-producing *Klebsiella pneumoniae* in companion animal infections in Portugal

Fröhlich K., Post-transcriptional control in the SOS response of *Klebsiella pneumoniae*

Braun H., Revisiting the unknown: the secretome of *K. pneumoniae*

Maranga M., Kleborate v3: Modular genotyping framework for *Klebsiella* species

Kochan T. J., Developing a multiple linear regression model to predict hypermucovisity in *Klebsiella pneumoniae*

Strysko J., Using comparative genomics to detect clusters of circulating *Klebsiella pneumoniae* among hospitalized neonates: Botswana, 2022-2024

Lam M., Genomic insights around AMR and hypervirulence convergence in a global dataset of *Klebsiella pneumoniae*

Winkler M. A., Plasmid diversity in Norwegian *Klebsiella pneumoniae* isolates

Stanton T., Next-generation serotyping of *Klebsiella pneumoniae* for antigen prediction and vaccine development

Bruchmann S., Pangenome, Essential Gene and Transcriptome Analysis of *Klebsiella pneumoniae* Reveal Key Lineage-Specific Genes

Tsang K., Ciprofloxacin resistance in *Klebsiella pneumoniae*: phenotype prediction from genotype

5:50-6:50 pm - Session 2: BMGF session: *Klebsiella pneumoniae* in LMICs: the path toward a maternal vaccine

Chairs: Usman Nakakana, Bill & Melinda Gates Foundation, United States - Nicole Benson, Bill & Melinda Gates Foundation, United States

- *Klebsiella pneumoniae* as an important infectious cause of death in infants in LMIC settings and the potential value of a maternal vaccine

  Invited speaker: **Ziyaad Dangor** (Chris Hani Baragwanath Academic Hospital, University of the Witwatersrand, Johannesburg, South Africa)

- Current insights on the development of a *Klebsiella pneumoniae* vaccine

  Invited speaker: **Francesca Micoli** (GSK Vaccines Institute for Global Health, Siena, Italy)

- Current progress of Inventprise's glycoconjugate vaccine development against *Klebsiella pneumoniae* for prevention of neonatal sepsis

  Invited speaker: **Heather Fox** (Inventprise Inc., Redmond, WA, United States)

6:50-7:10 pm - Poster pitches

Chair: Chiara Crestani, Institut Pasteur, France

Reddy D., Clinical and Molecular Epidemiology of *Klebsiella pneumoniae* Invasive Disease in Adults: preliminary results from a South African case-control study

Medina Cruz V. A., Epidemiological genomics of clinical isolates carbapenem-resistant and No-resistant *Klebsiella pneumoniae* related to healthcare-associated infections in Colombia 2020-2021

Planet P., Genomic epidemiology of *Klebsiella pneumoniae* in a hospital in Southern Africa, 2021-2024

Sierra R., Emergence of a triple carbapenemase broad host range IncC plasmid bearing blaKPC-2, blaNDM-1 and blaVIM-24

Tan S. M., Rational design of live bacterial therapeutics to clear *Klebsiella pneumoniae* from the gut

Soares Do Nascimento T., *Klebsiella pneumoniae* infection modulates the prenylation of macrophage proteins

Vornhagen J., A systematic study to identify microbiome-dependent *Klebsiella pneumoniae* gut fitness factors

Lim C. F. H., Virulence plasmid determinants of hypervirulent *Klebsiella pneumoniae* confer varying pathogenicity in different models of infection

Koh A., Insights from a novel dual humanised mouse model to study the pathogenesis of *Klebsiella pneumoniae*

Hernandez G. E., Bottlenecks and dissemination patterns associated with hypervirulent *Klebsiella pneumoniae*

Micoli F., Investigating the role of capsular and subcapsular antibodies for a vaccine against neonatal sepsis caused by *Klebsiella pneumoniae*

Osbelt-Block L., Utilizing metabolic capabilities of No-pneumoniae *Klebsiella species* to eradicate multi-drug resistant *Klebsiella pneumoniae* from the gut

Mullins L., Production, characterization and immunization with *Klebsiella pneumoniae* outer membrane vesicle vaccines containing genetically detoxified lipopolysaccharide

Sünderhauf D., Abundant gene transfer from *Klebsiella pneumoniae* clinical isolates to *Escherichia coli* can be prevented using CRISPR-Cas9.

7:10-9:30 pm - Poster session

<u>Thursday, November 21st, 2024</u>

9:00-10:30 am - Session 3: Ecology and One Health

Chairs: Edward Feil, University of Bath, United Kingdom - Iren Høyland Löhr, Stavanger University Hospital, Norway

- *Klebsiella pneumoniae* epidemiology through a One Health lens: Genomic insights into population dynamics across human, animal, and marine sources in Norway

  Invited speaker: **Marit Hetland** (Stavanger University Hospital, Stavanger, Norway)

- Genomic Diversity, Virulence, and Antimicrobial Resistance Profiles of *Klebsiella pneumoniae* in Madagascar: A One Health Approach

  Selected abstract: **Lala Rafetrarivony** (Experimental Bacteriology, Pasteur Institute of Madagascar, Antananarivo, Madagascar)

- Emergence of carbapenem-resistant *Klebsiella pneumoniae* isolates in French companion animals

  Selected abstract: **Marisa Haenni** (Unité Antibiorésistance et Virulence Bactériennes, Université de Lyon - Anses, Lyon, France)

- Class A beta-lactamases are core genes of the *Klebsiella planticola* and *Klebsiella terrigena* species complexes

  Selected abstract: **Lesley Hoyles** (Biosciences, Nottingham Trent University, Nottingham, United Kingdom)

- Deciphering phylogenetic and acquired drivers of ecological adaptation in the *Klebsiella pneumoniae* species complex

  Selected abstract: **Jose F Delgado-Blas** (Biodiversity and Epidemiology of Bacterial Pathogens, Institut Pasteur, Université de Paris, Paris, France)

11:00 am-12:30 pm - Session 4: *Klebsiella* molecular biology

Chairs: Guido Werner, Robert Koch Institute, Germany - Olaya Rendueles, CNRS, France

- Single amino acid polymorphism dictates the offensive versus defensive type VI secretion system behaviour affecting horizontal gene transfer in *Klebsiella pneumoniae.*

  Selected abstract: **Alix Lee** (Queen's University Belfast, Belfast, United Kingdom)

- RNA-RNA interactome of hypervirulent *K. pneumoniae* reveals a small RNA inhibitor of capsular mucoviscosity and pathogenesis in mice

  Selected abstract: **Yanjie Chao** (Shanghai Insititute of Immunity and Infection, Former Institut Pasteur of Shanghai, Chinese Academy of Sciences, Shanghai, China)

- Dynamics of *Klebsiella pneumoniae* bacteremia

  Invited speaker: **Michael Bachman** (Department of Pathology, University of Michigan Medical School, Ann Arbor, Michigan, United States Department of Microbiology and Immunology, University of Michigan Medical School, Ann Arbor, Michigan, United States)

- *K. pneumoniae* K64 capsule serotype: a facilitator of hypervirulence and multidrug resistance convergence?

  Selected abstract: **Julie Le Bris** (Microbial Evolutionary Genomics Unit, Department of Genomes & Genetics, Institut Pasteur, Paris, France Collège Doctoral, Ecole Doctorale Complexité du Vivant, Sorbonne Université, Paris, France)

- Discrete environmental cues regulating *Klebsiella pneumoniae* mucoidy

  Selected abstract: **Laura Mike** (Dept of Medicine, Division of Infectious Diseases, University of Pittsburgh, Pittsburgh, United States University of Toledo, Toledo, United States)

2:00-3:30 pm - Session 5: Host-pathogen interactions

Chairs: Christiane Forestier, Université Clermont Auvergne, France - Margaret Lam, Monash University, Australia

- *Klebsiella* goes on the offensive: T6SS-dependent manipulation of vascular physiology

  Invited speaker: **José Bengoechea** (Queen's University Belfast, Belfast, United Kingdom)

- *Klebsiella pneumoniae* SEFIR domain encoded effector - a new immune evasin

  Selected abstract: **Joana Sa Pessoa** (Wellcome-Wolfson Institute for Experimental Medicine, Queen's University Belfast, Belfast, United Kingdom)

- Differential myeloid-pathogen interactions define unique immune pathology in the respiratory tract of classical and hypervirulent *Klebsiella pneumoniae* infection

  Selected abstract: **Teck Hui Teo** (Mucosal infection lab, A*STAR ID Labs, Singapore, Singapore)

- Phase variation of mucoid capsule in hypervirulent *Klebsiella pneumoniae*

  Selected abstract: **Joseph Wanford** (King's College London, London, United Kingdom)

- Seeing what sticks: how hypermucoviscosity-associated *rmp* genes impact classical *Klebsiella pneumoniae* virulence

  Selected abstract: **Stephen Salisbury** (Micro & Immuno, Virginia Miller lab, University of North Carolina - Chapel Hill, Chapel Hill, United States)

4:00-5:30 pm - Session 6: Infection models Genomics and evolution

Chairs: José Bengoechea, Queen's University Belfast, United Kingdom - Yunn-Hwen Gan, National University of Singapore.

- *Klebsiella pneumoniae*: silent colonizer to rowdy invader

  Invited speaker: **Ammar Zafar** (Wake Forest University School of Medicine, Winston-Salem, United States)

- Application of various murine models to Probe *Klebsiella pneumoniae* pathogenesis, host immunity, and therapeutic efficacy

  Invited speaker: **David Rosen** (Department of Pediatrics, Washington University, St. Louis, United States)

- Antibiotic treatment failure of liver abscesses caused by hypervirulent *Klebsiella pneumoniae*

  Selected abstract: **Sarah Rowe-Conlon** (Microbiology and Immunology, University of North Carolina at Chapel Hill, Chapel Hill, United States)

- *In vivo* single-cell high-dimensional mass cytometry analysis to track the interactions between *Klebsiella pneumoniae* and myeloid cells

  Selected abstract: **Ricardo Calderón González** (Wellcome-Wolfson Institute For Experimental Medicine, Queen's University Belfast, Belfast, United Kingdom)

5:30-6:30 pm - Poster session

Friday, November 22nd, 2024

9:00-11:00 am - Session 7: Genomics and evolution

Chairs: Kelly Wyres, Monash University, Australia - Sylvain Brisse, Institut Pasteur, France

- Delivering genomic interpretation for local value, and towards national and international policy

  Invited speaker: **David Aanensen** (Oxford University, Oxford, United Kingdom)

- The extracellular capsule: Evolution and consequences for *K. pneumoniae*

  Invited speaker: **Olaya Rendueles** (Institut Pasteur, Université Paris Cité, CNRS UMR3525, Microbial Evolutionary Genomics, Paris, France Laboratoire de Microbiologie et Génétique Moléculaires (LMGM), CNRS UMR5100, Centre de Biologie Intégrative (CBI), Université de Toulouse, CNRS, Université Toulouse III - Paul Sabatier (UT3), Toulouse, France)

- A metabolic atlas of the *Klebsiella pneumoniae* Species Complex

  Selected abstract: **Ben Vezina** (Department of Infectious Diseases, Monash University, Melbourne, Australia Centre to Impact AMR, Monash University, Clayton, Australia)

- Plasmidomics of a global collection of *Klebsiella pneumoniae* reveals diverse routes for the convergence of resistance and virulence genes

  Selected abstract: **Melissa Martin** (Multidrug Resistant Organism Repository and Surveillance Network, Walter Reed Army Institute of Research, Silver Spring, United States)

- Deciphering transmission patterns of NDM-1 producing *Klebsiella pneumoniae*: Spread of different NDM-1 carrying plasmids across four different hospital sites in Berlin, Germany

  Selected abstract: **Anna Weber** (Institute of Hygiene and Environmental Medicine, Charité - Universitätsmedizin Berlin, Berlin, Germany)

- The role of genetic background on the emergence and spread of antibiotic resistance in *Klebsiella pneumoniae*

  Selected abstract: **Evan Snitkin** (Microbiology and Immunology, University of Michigan, Ann Arbor, United States)

11:30 am-1:00 pm - Session 8: Vaccines and Novel therapeutic approaches

Chairs: Michael Bachman, Michigan University, United States - Kat Holt, London School of Hygiene & Tropical Medicine, United Kingdom

- Phage therapy targets IBD-driving *K. pneumoniae* in the gut

  Invited speaker: **Sara Federici** (Weizmann Institute of Science, Rehovot, Israel)

- A multi-valent *Klebsiella pneumoniae* capsule vaccine

  Selected abstract: **Paeton Wantuch** (Pediatric Infectious Disease, Washington University School of Medicine, Saint Louis, Mo, United States)

- K-Vax, a new vaccine for prevention of disease by *Klebsiella pneumoniae* with potential for global strain coverage

  Selected abstract: **Juan Jose Infante** (VAXDYN, Dos Hermanas, Spain)

- Anti-capsule human monoclonal antibodies protect against hypervirulent and pandrug-resistant *Klebsiella pneumoniae*

  Selected abstract: **Anna Kabanova** (Fondazione Toscana Life Sciences, Siena, Italy)

- A community-driven resource for *Klebsiella* research identified a novel gut phage signature associated with chronic disease

  Selected abstract: **Daniela Rothschild Rodriguez** (University of Southampton, Southampton, United Kingdom)

1:00-2:00 pm - Panel discussion

Moderator: Padmini Srikantiah, Bill & Melinda Gates Foundation, United States

Topic: How do you solve a problem like *Klebsiella*? Focus on surveillance and infection control

Invited speakers: Michael Bachman (Michigan University, Ann Arbor, United States), Amy Mathers (University of Virginia School of Medicine, Charlottesville, United States), Kat Holt (London School of Hygiene & Tropical Medicine, London, United Kingdom), Keith Klugman (Bill & Melinda Gates Foundation, Bonita Springs, United States), Shabir Madhi (Vaccines and Infectious Diseases Analytics Research Unit, University Of The Witwatersrand, Johannesburg, South Africa)
